# Traumatic Childbirth Experience and Postnatal Post-traumatic Stress Disorder

**DOI:** 10.1192/j.eurpsy.2025.2426

**Published:** 2025-08-26

**Authors:** B. Peixoto, M. Cruz, V. Ustares

**Affiliations:** 1 Psychiatry, Hospital do Divino Espirito Santo; 2 Psychiatry, Hospital do Divino Espírito Santo, Ponta Delgada, Portugal

## Abstract

**Introduction:**

Although childbirth represents a positive experience for most mothers and fathers, it can also be potentially traumatic when it endangers the life of the mother or the baby. In more severe cases, it can even develop into Post-Traumatic Stress Disorder (PTSD) postpartum. Studies indicate birth-related PTSD impacts around 17% of postpartum parents. This condition includes intrusive symptoms, hyperactivation, avoidance behaviors, and negative changes in mood and cognition, significantly impacting the mother-baby bond and the family’s well-being.

**Objectives:**

The authors pretend to raise awareness of postpartum PTSD.

**Methods:**

The authors did a non-systematic review of the current literature.

**Results:**

The etiology of postpartum PTSD is multifactorial and results from the combination of pre-birth risk factors (depression during pregnancy, fear of childbirth, medical complications during pregnancy, history of trauma or sexual abuse, history of mental disorders), factors during childbirth (subjectively experienced negative childbirth, obstetric complications, and severe maternal morbidity), and postpartum factors (postpartum depression, maternal complications after childbirth, or maladaptive coping mechanisms). The risk factors related to childbirth appear to be independent of neonatal complications, with the latter constituting an additional stressor factor. Psychological trauma during childbirth and postpartum PTSD, despite having a significant impact on families, are often not recognized in maternity services, hindering timely intervention. Currently, there are no recommended treatments to prevent or mitigate postpartum PTSD. However, there is some evidence of the benefits of prenatal and postnatal interventions, such as early identification of risk factors for postpartum PTSD and postnatal counseling.

**Conclusions:**

The studies suggests that women should be assessed for negative traumatic birth experiences and PTSD, to allow targeted observation for psychopathologies and therapeutic interventions. Further research is needed with larger sample sizes, validated and reliable clinical interviews to assess PTSD.

**Disclosure of Interest:**

None Declared

